# Supplementary material for: RagD auto-activating mutations impair MiT/TFE activity in kidney tubulopathy and cardiomyopathy syndrome
Source: Nat Commun. 2023 May 15;14:2775. doi: 10.1038/s41467-023-38428-2 (PMC10185561; doi:10.1038/s41467-023-38428-2)
Supplement: Supplementary file 1 — Supplementary Information [file 41467_2023_38428_MOESM1_ESM.pdf]

Supplementary information

RagD auto-activating mutations impair MiT/TFE activity in kidney tubulopathy and cardiomyopathy syndrome

Irene Sambri[1,2*], Marco Ferniani[1,2*], Giulia Campostrini[3], Marialuisa Testa[1], Viviana Meraviglia[3], Mariana E. G. de Araujo[4], Ladislav Dokládal[5], Claudia Vilardo[1], Jlenia Monfregola[1], Nicolina Zampelli[1], Francesca Del Vecchio Blanco[6], Annalaura Torella[1,6], Carolina Ruosi[7], Simona Fecarotta[2], Giancarlo Parenti[1,2], Leopoldo Staiano[1,8], Milena Bellin [3,9,10], Lukas A. Huber[4], Claudio De Virgilio[5], Francesco Trepiccione[7,11], Vincenzo Nigro[1,6], Andrea Ballabio[1,2,12,13]

1 Telethon Institute of Genetics and Medicine (TIGEM), Pozzuoli (NA), Italy.
2 Medical Genetics Unit, Department of Medical and Translational Science, Federico II University, Naples, Italy.
3 Department of Anatomy and Embryology, Leiden University Medical Center, 2333ZC Leiden, the Netherlands
4 Institute of Cell Biology, Biocenter, Medical University of Innsbruck, Innsbruck, Austria.
5 Department of Biology, University of Fribourg, CH-1700 Fribourg, Switzerland.
6 Department of Precision Medicine, University of Campania "Luigi Vanvitelli", Naples, Italy
7 Department of Translational Medical Sciences, University of Campania "L. Vanvitelli", Naples, Italy.
8 Institute for Genetic and Biomedical Research, National Research Council (CNR), Milan, Italy.
9 Department of Biology, University of Padua, 35131 Padua, Italy
10 Veneto Institute of Molecular Medicine, 35129 Padua, Italy
11 Biogem Research Institute Ariano Irpino, Ariano Irpino, Italy.
12 Department of Molecular and Human Genetics, Baylor College of Medicine, Houston, TX, USA.
13 Jan and Dan Duncan Neurological Research Institute, Texas Children's Hospital, Houston, TX, USA.

* These authors contributed equally.

Correspondence to: Andrea Ballabio (ballabio@tigem.it)

Detailed clinical description of affected individuals.

We collected data from a wide range of ages (from 5 to 62 y/o) covering three generations (Figure 1C). The index patient (IV.8) was referred to our outpatient clinic for a rare tubulopathy. She was found hypokalemic at a laboratory check performed after her mother (III.15) died from sudden cardiac death. The patient presented hypomagnesemia secondary to increased renal loss of magnesium, evident both at fasting and after meal. Her clinical history included a diagnosis of normo-calciuric, normo-citraturic and normo-oxaluric calcium phosphate nephrolithiasis that manifested from the age of 5. Medullary nephrocalcinosis, mitral insufficiency and arrhythmias completed the clinical evaluation (Supplementary Table1). All these findings were also diagnosed to her mother (from the age of 6). Analysis of magnesium was not available from her mother laboratory records. Similar electrolyte values were present in her younger brother (IV.9). However, the analysis of his stones, collected by spontaneous passage at different ages (7 and 10 y/o), revealed that they were made of calcium oxalate. Finally, patient IV.9 did not show any heart disease as evaluated by 24h-Holter ECG monitoring and heart ultrasound. The whole pedigree evaluation, inheritance pattern (autosomal dominant) and high familial prevalence of heart disease questioned the diagnosis of the most common salt-losing nephropathies and required a genetic confirmation through WES. None of the patients was born pre-term or complained of any symptoms during infancy. Polydramnios was never reported. All patients had low levels of serum magnesium (0.64 ± 0.04 vs 0.80 ± 0.03 mM; mean ± sem; n 6:5; p-value < 0.01) and mild hypokalaemia (3.37 ± 0.22 vs 4.18 ± 0.09 mM; mean ± sem; n 7:5; p-value < 0.05) compared to non-affected family members. Muscle cramps, paresthesia and tetany were associated with these electrolyte abnormalities. Mild metabolic alkalosis and normotension were present in all affected family members. Serum potassium level increased following potassium sparing diuretics (first spironolactone and later eplerenone) treatment with no associated worsening of blood pressure as prescribed for DCM in patients III.2. Medullary nephrocalcinosis was present in 4/7 affected members, apparently not linked to hypercalciuria. All patients presented normal eGFR, including the two oldest (II-1 and II-7, aged 57 and 62 at last follow-up, respectively), suggesting that the renal disease did not progress towards kidney failure at this age. Sensorineural hypoacusia was not observed in any of the patients. The spectrum of heart disease in our cohort of patients was not restricted to DCM, but included also myocardial infarction, mitral valve insufficiency and arrhythmias. Asymptomatic ventricular bigeminy occurred earlier in life in most of our patients (5/7), including those who developed DCM (Supplementary Table 1). DCM required the implantation of an ICD device in both affected patients. However, none of them received a heart transplantation during infancy, even though patient III.2 is on the waiting list at age 42.

Supplementary Table 1

Patients'clinical and laboratory findings

ID			II.7	II.1	III.15	IV.8	IV.9	III.5	III.2
Gender			F	F	F	F	M	M	F
Age at manifestation	years		50	52	6	15	5	30	39
Current Age	years		63	59	35*	17	10	34	42
Heart diseases	type		V/Arr	DCM	V/Arr	V/Arr	NO	MI	DCM
Ejection fraction	%		60	38	45	-	-	45	25
NYHA class			NA	3	NA	NA	NA	NA	2
LVEDD	mm		42	62	49	-	-	54	67
ICD	Age at Implant		NO	55	NO	NO	NO	NO	39
Heart Transplant			NO	NO	NO	NO	NO	NO	WL
Hypoacusia			YES	YES	NO	NO	NO	NO	NO
HypoMg- Symptoms			YES	YES	YES	YES	NO	YES	YES
Nephrocalcinosis			NO	NO	YES	YES	YES	NO	YES
Nephrolithiasis	Stone type		NO	NO	Ca-pho	Ca-pho	Ca-Ox	NO	-
Polyuria			NO	NO	YES	NO	NO	NO	NO
SBP	mmHg		140	110	105	104	110	120	100
DBP	mmHg		70	65	75	75	70	80	60
Laboratory Findings	Unit	n. v.							
Age	years		62	57	26	15	8	33	37
eGFR	$ml/min/m^2$		96	95	64	111	97	117	55
$S\text{-}Na^+$	mM	135 - 145	143	139	138	138	137	137	136
$S\text{-}K^+$	mM	3.5 - 5	3.0	3.2	2.5	3.3	3.6	3.6	4.4
$S\text{-}Cl^-$	mM	98 - 107	103	107	103	104	105	101	104
$S\text{-}Ca^{2+}$	mM	2.20 - 2.65	2.35	2.42	2.7	2.37	2.25	2.04	2.7
$S\text{-}Mg^{2+}$	mM	0.7 - 1.1	0.53	0.60	-	0.74	0.78	0.60	0.58
$S\text{-}PO_4^-$	mM	0.6 - 1.4	1.25	1.01	1.42	1.00	1.42	0.96	1.35
$S\text{-}HCO_3^-$	mM	22 - 26	30	28	23	26	25	26	24
$Fe\text{-}Na^+$	%	< 1	0.42	0.80	1.99	0.37	0.30	-	-
$Fe\text{-}K^+$	%	5- 15	12	29	24	8	5	-	-
$Fe\text{-}Mg^{2+}$	%	< 4	4.25	5.04	-	4.15	5.86	-	-
$Ca^{2+}/Creat$	mmol/mmol	0.04 - 0.7	0.33	0.25	-	0.15	0.22	-	-

Supplementary Table 1

Clinical and laboratory findings of affected individuals from the family carrying the P88L *RRAGD* mutation: asterisk (*) indicates the age at which sudden cardiac death occurred. LVEDD = Left Ventricular End-Diastolic Diameter. V/arr = ventricular arrhythmia, DCM = dilated cardiomyopathy and MI = myocardial infarction. ICD = Implantable Cardiac Defibrillator. SBP and DBP = Systolic and Diastolic Blood Pressure. eGFR (estimated Glomerular Filtration Rate) was calculated by CKD-EPI and Swartz equations for patients > or < 18 y/o, respectively.

Supplementary Table 2

RagD		RagC				
Residue	2q3f GTP	Residue	6CES Free	6U62 GDP	6S6A GDP	6S6D GDP
P88A	0.34	P87A	0.29	--	--	0.43
P88L	0.87	P87L	0.06	--	--	0.83
P88R	0.82	P87R	0.03	--	--	1.04
P119A	1.06	P118A	-0.82	-0.81	--	--
P119L	0.55	P118L	-0.72	-0.80	--	--
P119R	1.16	P118R	-0.78	-0.85	--	--
I221A	2.62	I220A	0.12	1.69	1.30	2.14
I221P	2.69	I220P	1.13	0.97	1.02	1.59
I221K	1.18	I220K	-1.51	1.36	1.44	1.22

Supplementary Table 2.

● Protein stability

Prediction analysis of the impact of RagD amino acid mutations on RagD protein stability. The analysis was extended to the corresponding amino acid mutations in RagC. Patient relevant mutations are highlighted in grey. The table indicates the corresponding Gibbs binding free energy values given as kCal/mol.

Supplementary Table 3

PDB	Residue	WT	Mut	Δ/ΔG binding	ΔGbind WT	ΔGbind Mut
2q3f	76	Ser	Ala	-4.59	-65.66	-70.25
2q3f	76	Ser	Leu	-3.37	-65.66	-69.03
2q3f	76	Ser	Trp	-11.87	-65.66	-77.52
2q3f	88	Pro	Ala	-0.27	-65.66	-65.93
2q3f	88	Pro	Arg	-1.16	-65.66	-66.82
2q3f	88	Pro	Leu	-0.36	-65.66	-66.02
2q3f	97	Thr	Ala	-4.00	-65.66	-69.66
2q3f	97	Thr	Pro	6.32	-65.66	-59.34
2q3f	97	Thr	Lys	-13.62	-65.66	-79.28
2q3f	119	Pro	Ala	-0.62	-65.66	-66.28
2q3f	119	Pro	Leu	-0.79	-65.66	-66.45
2q3f	119	Pro	Arg	-2.62	-65.66	-68.28
2q3f	221	Ile	Ala	5.12	-65.66	-60.54
2q3f	221	Ile	Pro	25.61	-65.66	-40.04
2q3f	221	Ile	Lys	-6.89	-65.66	-72.55

Supplementary Table 3.

Prediction analysis of the impact of RagD amino acid mutations on the binding to GTP. Patient relevant mutations are highlighted in grey. Gibbs binding free energy values are given as kCal/mol.

Supplementary Figure1

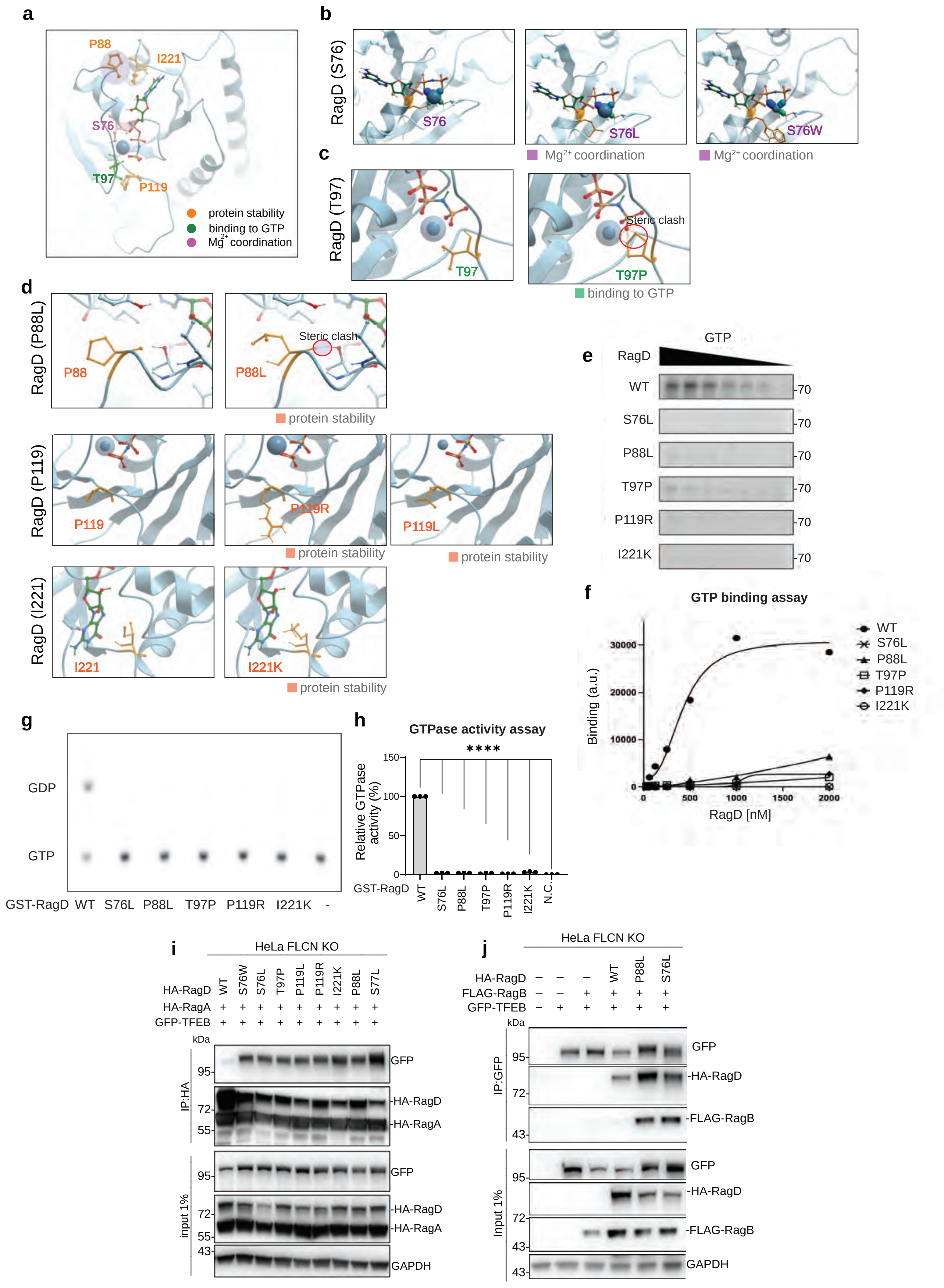

Supplementary Figure 1. ***In silico*** **modeling and in vitro assays of RagD mutations.**

(**a**) Position of the residues in the RagD GTP according to RagD structure in Protein Data Bank (PDB code 2q3f), color coded according to the predicted effects of the respective mutations. (**b**) Zoomed views of S76 and the *in silico* models of the respective mutations are shown (S76L/W). Both S76L and S76W display defects in Mg^{2+} coordination (annotated in magenta) (**c**) Zoomed view of RagD T97 and the *in silico* model of T97P are shown. The model could not resolve local steric clashes (annotated as a red circle). For T97P the binding to GTP is annotated in green. (**d**) Zoomed views of P88, P119 and I221 and the *in silico* models of the respective mutations P88L, P119R/L and I221K are shown (annotated in orange). For P88L, the model could not resolve a local steric clash (annotated as a red circle). All patient mutations in this group were predicted to have decreased stability when bound to GTP. See also Supplementary Table 2. (**e**) Binding of [α-^{32}P]-GTP to indicated recombinant GST-RagD variants (WT, S76L, P88L, T97P, P119R, I221K) visualized by autoradiography (see methods section for details). (**f**) Quantification of radioactive signals using ImageJ and the binding curve was obtained by sigmoidal nonlinear regression (4PL) with an interpolation from a standard curve in a confidence interval of 95% using Prism 9 software (Graphpad). (**g**) TLC separation of radiolabeled GDP from non-hydrolyzed GTP. The initial buffer containing [α-^{32}P]-GTP was spotted in the last lane. The conversion of GTP to GDP by WT RagD protein is shown in lane 1. (**h**) Relative quantification of the radiolabeled signal of TLC separation showed in **g** (n=3 independent experiment). Ordinary One-way ANOVA Tukey multiple comparison test (****$p < 0.0001$). (**i**) Immunoprecipitation performed in HeLa *FLCN* KO cells transfected with GFP-TFEB and HA-RagD WT or HA-RagD mutants (S76L/W, T97P, P119L, P119R, I221K, P88L, S77L) and with equimolar amount of HA-RagA WT. The HA tag was used as bait for the immunoprecipitation (n=2 independent experiments). (**j**) Immunoprecipitation performed in HeLa *FLCN* KO cells transfected with GFP-TFEB (bait) and HA-RagD WT or HA-RagD mutants (S76L, P88L) and with equimolar amount of FLAG-RagB WT (n=2 independent experiments).

Supplementary Figure 2

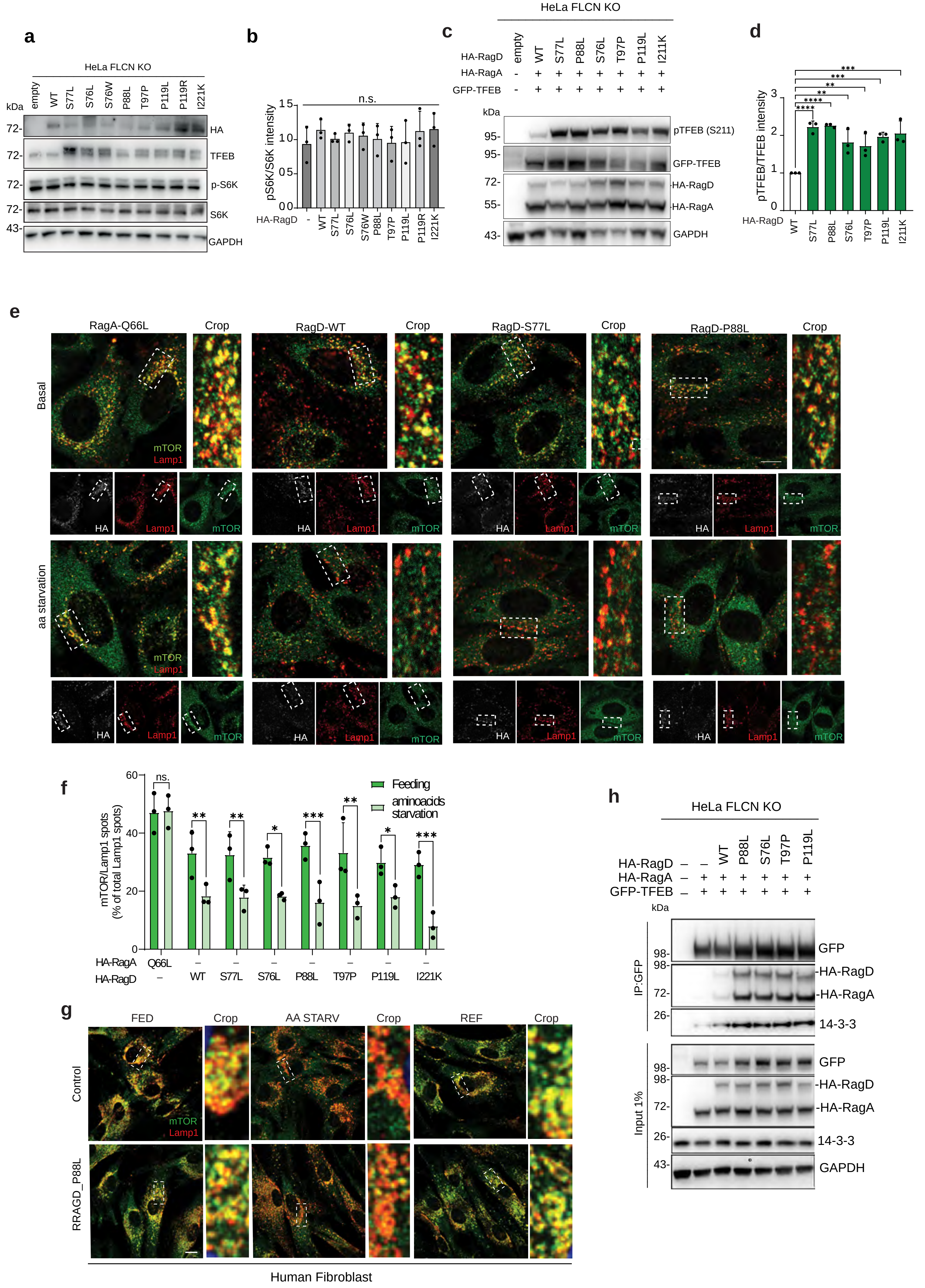

Supplementary Figure 2. RagD mutants do not affect the canonical mTORC1 activity.

(**a**) Representative western blot of cell lysates of HeLa *FLCN* KO cells transiently transfected with HA-RagD WT or HA-RagD mutants (S77L, S76L, S76W, P88L, T97P, P119L, P119R, I221K). anti-TFEB, anti-phospho-S6K, anti-S6K and anti-GAPDH were used to evaluate TFEB molecular weight shift as well as the phosphorylation status of S6K. (**b**) Graph represents the relative quantification of S6K phosphorylation shown in **a** (mean ± s.d. for n=3 independent experiments). Ordinary One-way ANOVA Dunnett's multiple comparison test (ns>0.9) (**c**) Representative western blot of cell lysates of HeLa *FLCN* KO cells transiently transfected with HA-RagD WT or HA-RagD mutants (S77L, P88L, S76L, T97P, P119L, I221K) and GFP-TFEB to monitor TFEB serine 211 phosphorylation (pTFEB S211 antibody). (**d**) Graph shows the relative quantification of TFEB S211 phosphorylation levels shown in **c** (mean ± s.d. for n=3 independent experiments). Ordinary One-way ANOVA Dunnett's multiple comparison test (**$p < 0.01$, ***$p < 0.001$, ****$p < 0.0001$). (**e**) Representative immunofluorescence images of Hela WT transiently transfected with HA-RagA-Q66L, HA-RagD-WT, HA-RagD S77L and HA-RagD P88L either untreated or subjected to amino acid (aa) starvation for 1 hour, immunostained with anti-HA, anti-Lamp1 and anti-mTOR antibodies. Scale bar, 10 μm. (**f**) Graph represents the percentage of mTOR/Lamp1 spots in HeLa cells transiently transfected with HA-RagD WT or mutants (S77L, S76L, P88L, T97P, P119L, I221K) either untreated or subjected to amino acids (aa) starvation for 1 hour. RagA-Q66L was used as positive control (mean ± s.d. of n > 1,000 cells from n=3 independent experiments). Quantification performed using the Perkin-Elmer Opera system (see Methods). Ordinary two-way ANOVA Sidak's multiple comparison test (ns>0.9 *$p < 0.05$, **$p < 0.01$, ***$p < 0.001$). (**g**) Immunofluorescence staining representing the mTOR lysosomal localization in normal feeding (FED), amino acid starved (AA STARV) and amino acid replenished conditions (REF) in human fibroblast carrying the *RRAGD* P88L mutation and relative control cells (n=3 independent experiments). Scale bar, 10 μm. (**h**) Immunoprecipitation performed in HeLa *FLCN* KO cells transfected with GFP-TFEB (bait) and HA-RagD WT or HA-RagD mutants (P88L, S76L, T97P, P119L) and with equimolar amount of HA-RagA WT. Western blot analysis shows the increased interaction between TFEB and 14-3-3 in presence of RagD mutants (n=2 independent experiments).

Supplementary Figure 3

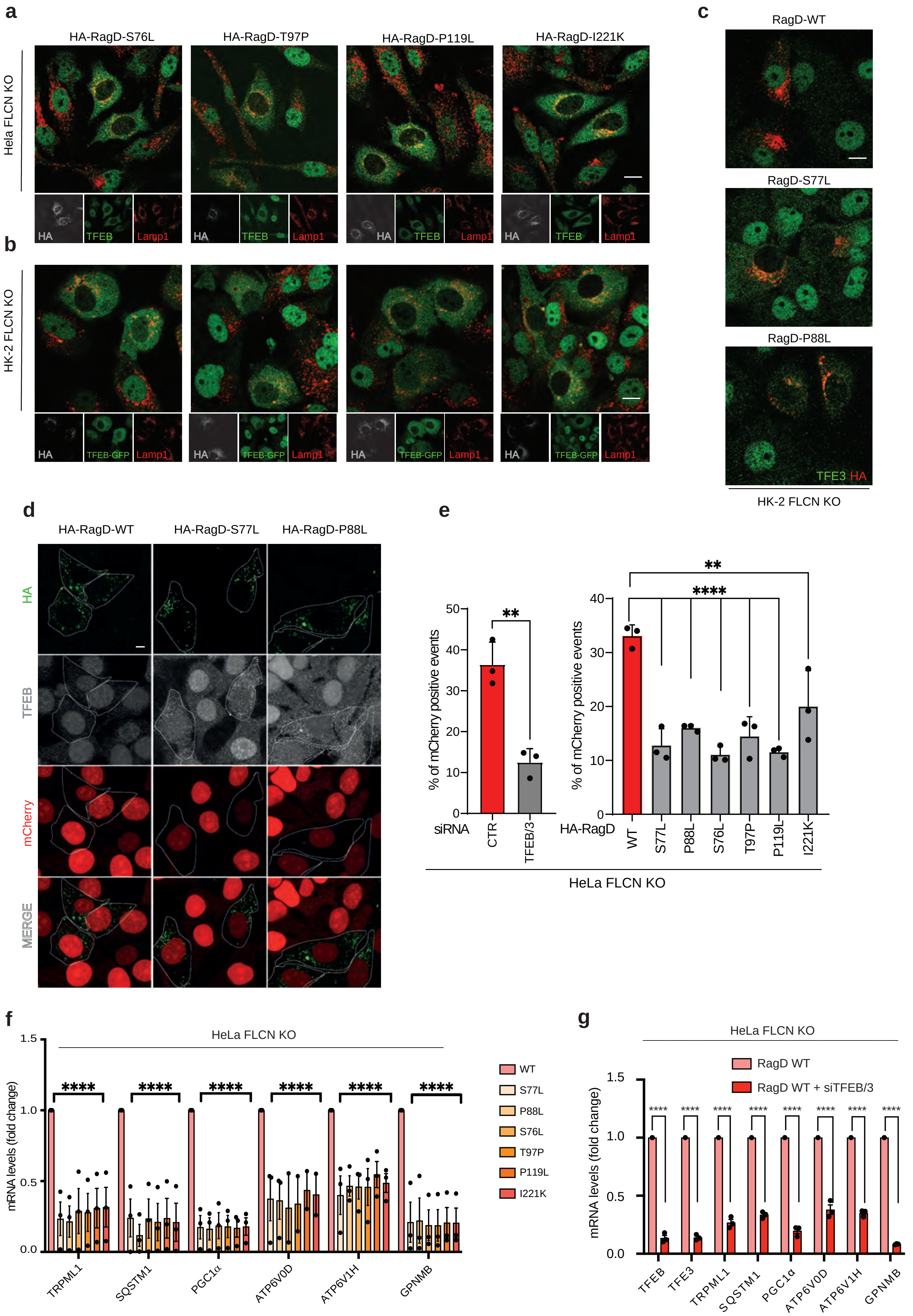

Supplementary Figure 3. RagD mutants impair TFEB transcriptional activity.

(**a**) Representative immunofluorescence images of TFEB, HA and Lamp1 antibodies staining in HeLa *FLCN* KO cells transiently transfected with HA-RagD mutants S76L, T97P, P119L and I221K (n=3 independent experiments). Scale bar, 10 µm. (**b**) Representative immunofluorescence images of TFEB, HA and Lamp1 antibodies staining in HK-2 *FLCN* KO cells carrying an inducible TFEB-GFP transiently transfected with HA-RagD mutants S76L, T97P, P119L and I221K (n=3 independent experiments). Scale bar, 10 µm. (**c**) Representative immunofluorescence images of TFE3 and HA antibody staining in HK-2 *FLCN* KO cells transiently transfected with HA-RagD-WT or HA-RagD mutants S77L and P88L (n=3 independent experiments). Scale bar, 10 µm. (**d**) Representative images of Hela *FLCN* KO cells stably expressing the GPNMBprom-mCherry reporter. Cells were transiently transfected with HA-RagD-WT or HA-RagD mutants (S77L, P88L) and immunostained with anti-HA and anti-TFEB antibodies (n=3 independent experiments). (**e**) Analysis of MiT-TFE transcriptional activity using a GPNMBprom-mCherry fluorescent transcriptional reporter. HeLa *FLCN* KO cells stably expressing the GPNMBprom-mCherry reporter were transiently transfected with siRNA TFEB and TFE3 or WT or mutated forms of HA-RagD. HA positive cells were isolated with an anti-HA antibody and mCherry fluorescence intensity analyzed by FACS. Results are mean ± sd; n=1000 HA-positive cells for each condition of n=3 independent experiment. Ordinary One-way ANOVA Dunnett's multiple comparison test was used on RagD WT vs RagD mutants. Two-tailed unpaired student t-Test was performed on siTFEB/3 vs siCTR. (**$p < 0.01$, ****$p < 0.0001$) (**f**) HeLa *FLCN* KO cells were transiently transfected with either RagD WT or mutants (S77L, P88L, S76L, T97P, P119L, I221K) and subjected to qRT–PCR. Relative mRNA levels of the indicated genes were normalized to levels of *HPRT1* and expressed as fold change relative to control samples. Results are mean ± SEM (n = 3 independent experiment). Linear regression was used for the presence of more than two groups. 'p-value' was corrected with Benjamini-Hochberg correction for multiple testing (****$p < 0.0001$). (**g**) Relative mRNA levels of the indicated genes in HeLa *FLCN* KO cells transiently transfected with RagD WT and/or depleted for TFEB and TFE3 using specific siRNAs. Results are mean ± SEM (n = 3 independent experiment). Statistical analysis performed as in **f**.

Supplementary Figure 4

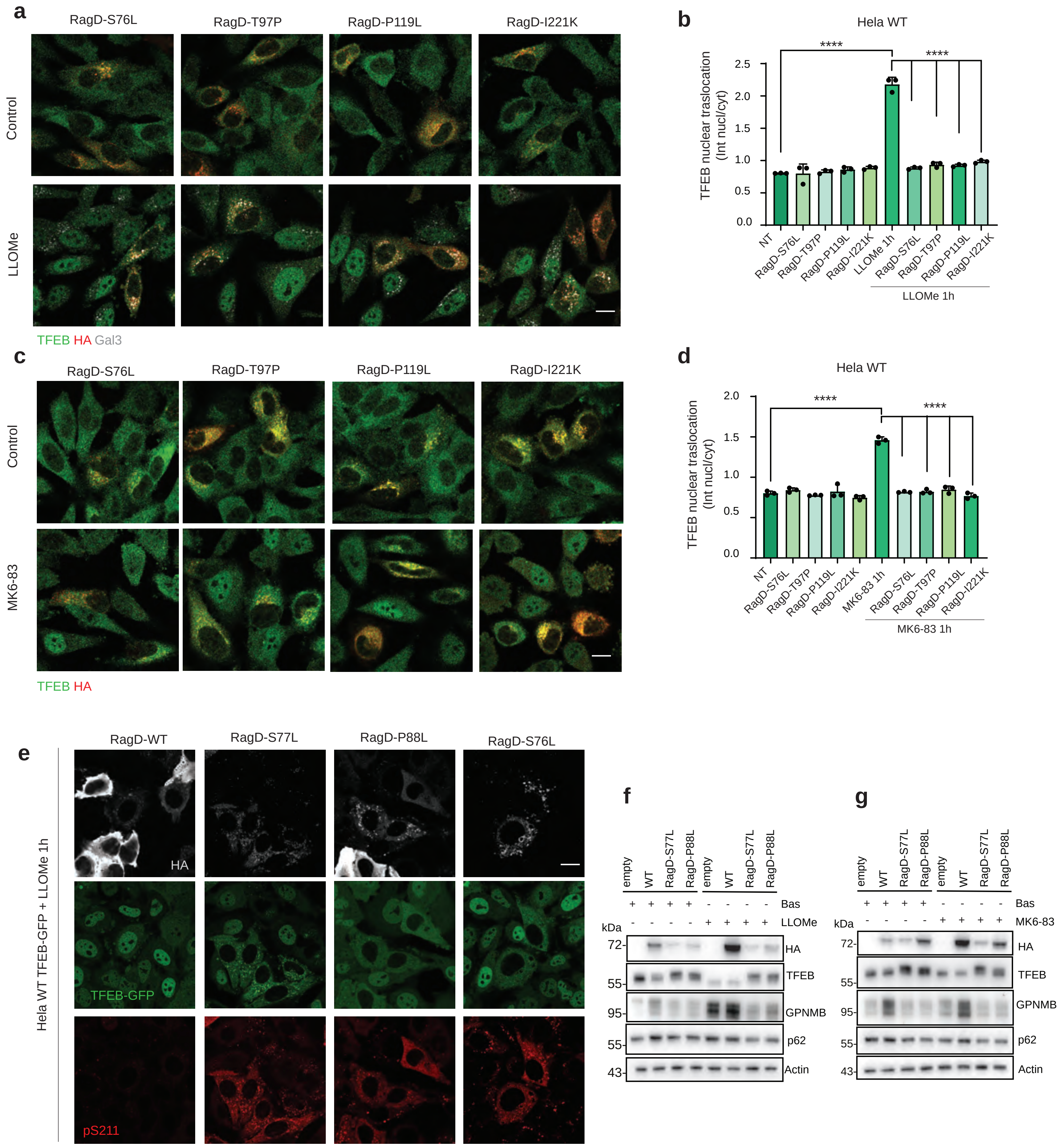

Supplementary Figure 4. RagD mutants retain TFEB in the cytosol upon lysosomal damaging agent treatments.

(**a**) Representative images of HeLa cells transiently transfected with HA-RagD mutants (S76L, T97P, P119L, I221K) and immunostained with anti-TFEB, anti-HA and anti-Galectin-3 (Gal3). Scale bar, 10 µm. Untreated control samples and samples treated with 500 µM LLOMe for 1 hour are shown. (**b**) Graph showing the TFEB nuclear localization following LLOMe treatment in HA-RagD mutants (S76L, T97P, P119L, I221K) positive HeLa cells (mean ± s.d. of n > 1,000 cells from n=3 independent experiments). Ordinary One-way ANOVA Tukey multiple comparison test (****$p < 0.0001$). (**c**) Representative images of HeLa cells transiently transfected with HA-RagD mutants (S76L, T97P, P119L, I221K) immunostained with anti-TFEB and anti-HA antibodies. Scale bar, 10 µm. Untreated control samples and samples treated with 30 µM MK6-83 for 1h are shown. (**d**) Graph showing the TFEB nuclear localization following MK6-83 treatment in HA-RagD mutants (S76L, T97P, P119L, I221K) positive HeLa cells (mean ± s.d. of n > 1,000 cells from n=3 independent experiments). Ordinary One-way ANOVA Tukey multiple comparison test (****$p < 0.0001$). (**e**) Immunostaining using TFEB S211 phospho-antibodies in HeLa TFEB-GFP cells transiently transfected with HA-RagD WT or mutants (S77L, P88L, S76L) and treated with 500 µM LLOME for 1 hour (n=2 independent experiments). Scale bar, 10 µm. (**f**) Western blot of HeLa cells transiently transfected with HA-RagD WT and mutants (S77L and P88L) treated with LLOMe for 6 hours to monitor the GPNMB and p62 protein expression levels (n=3 independent experiments). (**g**) Western blot of HeLa cells transiently transfected with HA- RagD WT and mutants (S77L and P88L) treated with MK6-83 for 1 hour to monitor the GPNMB and p62 protein expression levels (n=3 independent experiments).

Supplementary Figure 5

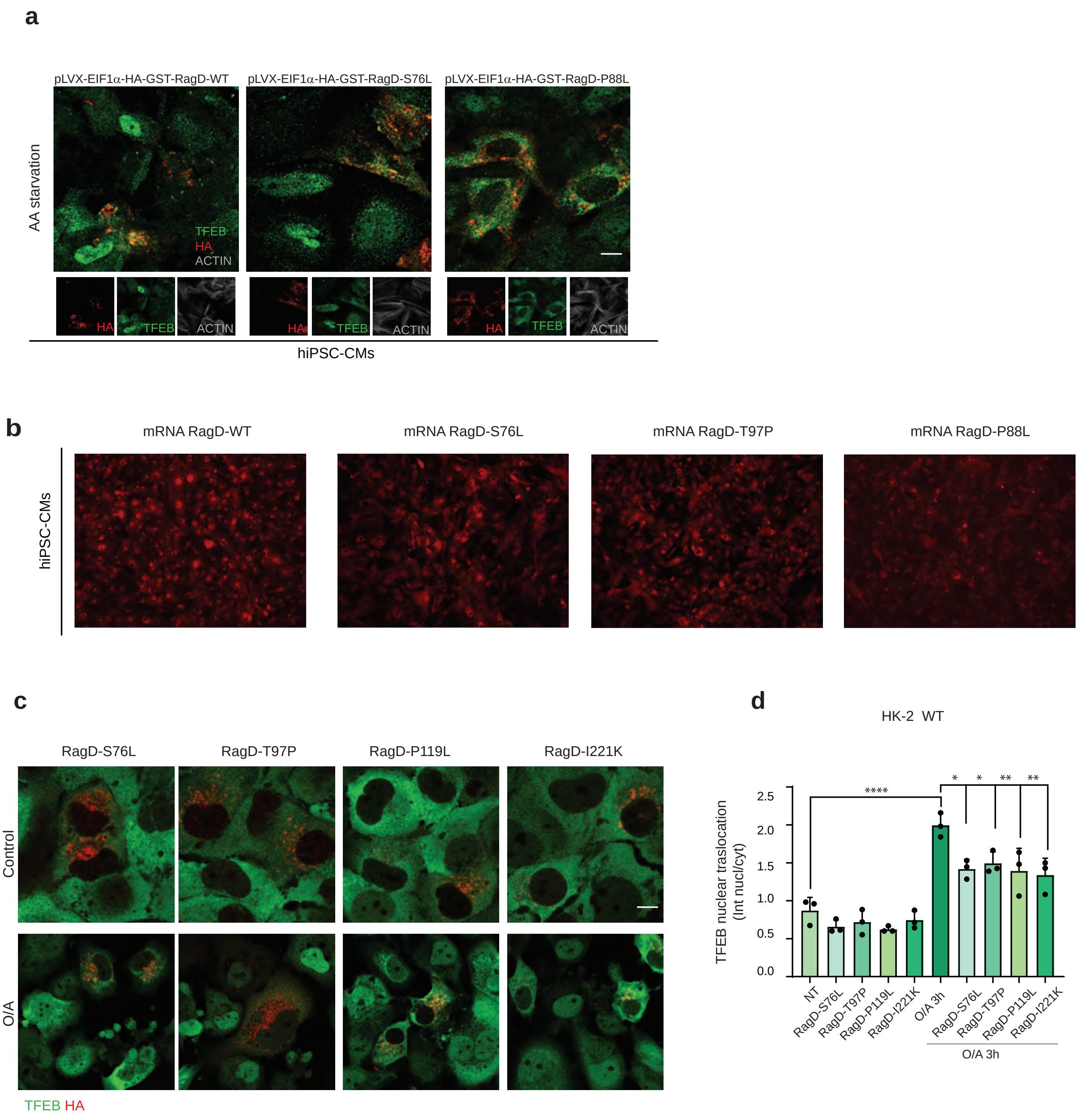

Supplementary Figure 5. RagD mutants cause TFEB cytosolic sequestration in starved hiPSC-CMs and in O/A treated HK-2 cells.

(**a**) Representative immunofluorescence in hiPSC-CMs infected with pLVX-EIF1a lentiviral vector carrying HA-RagD WT or RagD mutants (S76L, P88L). Anti-TFEB, anti-HA and anti-Actin were used for immunostaining (n=2 independent experiments). Scale bar, 10 µm. (**b**) Evaluation of mRNA-RagD-WT or -RagD mutants (S76L, P88L, T97P) transfection efficiency in hiPSC-CMs. Representative images showing mCherry positive cells 36h after transfection (n=2 independent experiments). Scale bar, 125 µm. (**c**) Representative images of HK-2 cells transiently transfected with HA-RagD mutants (S76L, T97P, P119L, I221K) either untreated or treated with 10 µg/ml Oligomycin and 5 µg/ml Antimycin A (O/A) for 3h immunostained with anti-TFEB and anti-HA antibodies. Scale bar, 10 µm. (**d**) Graph showing TFEB nuclear localization following O/A treatment in HA-RagD mutants (S76L, T97P, P119L, I221K) positive HK-2 cells (mean ± s.d. of n > 1,000 cells from n=3 independent experiments). Ordinary One-way ANOVA Tukey multiple comparison test (*$p < 0.05$, **$p < 0.01$, ****$p < 0.001$).

Supplementary Figures_Full Scans of western blots

Suppl Figure 1E

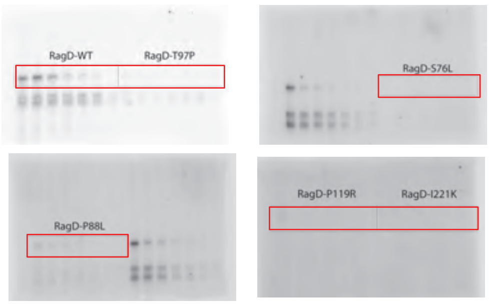

Suppl.Figure 1G

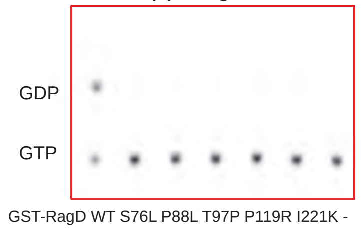

Suppl.Figure 1I

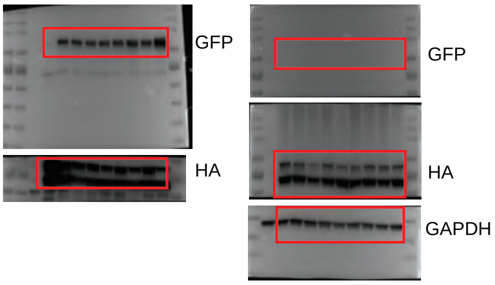

Suppl.Figure 2A

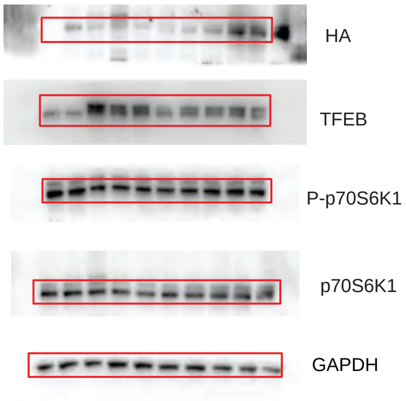

Suppl Figure 2C

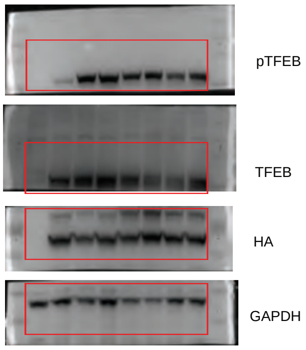

Suppl.Figure 1J

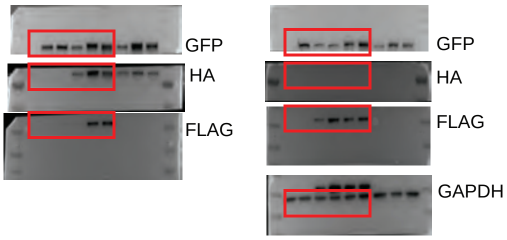

Suppl.Figure 2H

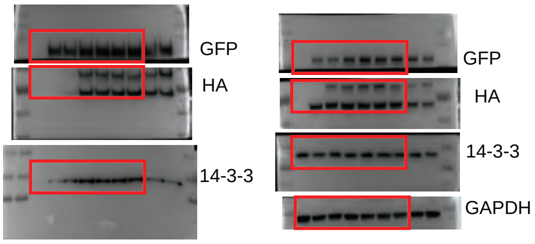

Suppl.Figure 4F

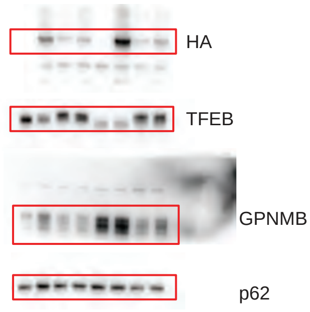

Suppl.Figure 4G

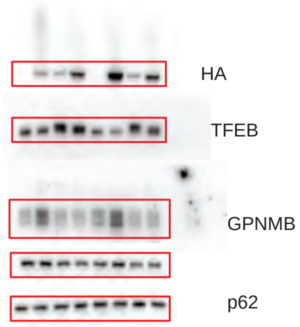

Supplementary Figure 6. Raw data WB Supplementary Figures.
