# Supplementary material for: RagD auto-activating mutations impair MiT/TFE activity in kidney tubulopathy and cardiomyopathy syndrome
Source: Nat Commun. 2023 May 15;14:2775. doi: 10.1038/s41467-023-38428-2 (PMC10185561; doi:10.1038/s41467-023-38428-2)
Supplement: Supplementary file 3 — Reporting Summary [file 41467_2023_38428_MOESM3_ESM.pdf]

nature portfolio

Corresponding author(s): Andrea Ballabio

Last updated by author(s): 2023/04/14

Reporting Summary

Nature Portfolio wishes to improve the reproducibility of the work that we publish. This form provides structure for consistency and transparency in reporting. For further information on Nature Portfolio policies, see our Editorial Policies and the Editorial Policy Checklist.

Statistics

For all statistical analyses, confirm that the following items are present in the figure legend, table legend, main text, or Methods section.

n/a	Confirmed	
☐	☒	The exact sample size (*n*) for each experimental group/condition, given as a discrete number and unit of measurement
☐	☒	A statement on whether measurements were taken from distinct samples or whether the same sample was measured repeatedly
☐	☒	The statistical test(s) used AND whether they are one- or two-sided<br>*Only common tests should be described solely by name; describe more complex techniques in the Methods section.*
☒	☐	A description of all covariates tested
☐	☒	A description of any assumptions or corrections, such as tests of normality and adjustment for multiple comparisons
☐	☒	A full description of the statistical parameters including central tendency (e.g. means) or other basic estimates (e.g. regression coefficient) AND variation (e.g. standard deviation) or associated estimates of uncertainty (e.g. confidence intervals)
☐	☒	For null hypothesis testing, the test statistic (e.g. *F*, *t*, *r*) with confidence intervals, effect sizes, degrees of freedom and *P* value noted<br>*Give P values as exact values whenever suitable.*
☒	☐	For Bayesian analysis, information on the choice of priors and Markov chain Monte Carlo settings
☒	☐	For hierarchical and complex designs, identification of the appropriate level for tests and full reporting of outcomes
☒	☐	Estimates of effect sizes (e.g. Cohen's *d*, Pearson's *r*), indicating how they were calculated

Our web collection on statistics for biologists contains articles on many of the points above.

Software and code

Policy information about availability of computer code

Data collection	Zeiss Zen Blue 2.1 software; Opera Phenix HCS system, Perkin Elmer.
Data analysis	For RagD mutations on protein stability and ligand binding: MERSi algorithm from Molsoft ICM-Pro 3.8-6a;<br>For experiments in cell lines: Graph pad Prism 8; Fiji 1.0; Microsoft Excel 2020; Columbus 2.6.0, Perkin Elmer.<br>For qPCR analysis: qpcR (version 1.4-1) available at the following url: https://cran.r-project.org/web/packages/qpcR/index.html (Ahmed et al., 2018. PMID: 29576953.

For manuscripts utilizing custom algorithms or software that are central to the research but not yet described in published literature, software must be made available to editors and reviewers. We strongly encourage code deposition in a community repository (e.g. GitHub). See the Nature Portfolio guidelines for submitting code & software for further information.

Data

Policy information about availability of data

All manuscripts must include a data availability statement. This statement should provide the following information, where applicable:

- Accession codes, unique identifiers, or web links for publicly available datasets
- A description of any restrictions on data availability
- For clinical datasets or third party data, please ensure that the statement adheres to our policy

Full scans for all western blots as well as raw data for all the graphs are provided with this manuscript as Supplementary Figure 6 and Source data file respectively.

For graphs the exact p value for all the experiments is present in the Source data file. The Whole-Exome Sequencing (WES) data are deposited in Sequence Read Archive (SRA) of NCBI repository (BioProject ID:PRJNA960632, available at the following link https://www.ncbi.nlm.nih.gov/bioproject/960632). All other data are available from the corresponding author on request.

Research involving human participants, their data, or biological material

Policy information about studies with human participants or human data. See also policy information about sex, gender (identity/presentation), and sexual orientation and race, ethnicity and racism.

Reporting on sex and gender	The term Gender (indicated in Table 1 as M and F) was used to indicate the biological attribute.
Reporting on race, ethnicity, or other socially relevant groupings	N/A
Population characteristics	The characteristics of the population involved in the study is exclusively based on the genotype (RRAGD gene variant) and on the presence of signs of the disease. Age range from 10 to 63 years old.
Recruitment	The human participants involved in the study belong to the same family and have been selected accordingly to their RRAGD genotype. No additional families have been found with the same mutation so no additional recruitment from other families has been possible.
Ethics oversight	*Identify the organization(s) that approved the study protocol.*

Note that full information on the approval of the study protocol must also be provided in the manuscript.

Field-specific reporting

Please select the one below that is the best fit for your research. If you are not sure, read the appropriate sections before making your selection.

☒ Life sciences ☐ Behavioural & social sciences ☐ Ecological, evolutionary & environmental sciences

For a reference copy of the document with all sections, see nature.com/documents/nr-reporting-summary-flat.pdf

Life sciences study design

All studies must disclose on these points even when the disclosure is negative.

Sample size	The sample size was chosen based on previous experience with respect to how many independent number of cells per treatment/group are required to reliably detect biologically meaningful differences among groups. Furthermore, most of the data have been carried out by High Content Imaging in which thousands of cells/group/treatement were analyzed. All the relevant information about sample size are reported in the figure legends.
Data exclusions	We did not apply any exclusion criteria
Replication	All experiments were carried out under standard and clearly defined conditions (based on our experience and on the literature), and were replicated successfully by at least one researcher. All attempts at replication were successful and the relative data are available in the Source Data File.
Randomization	The cells used in the manuscript were randomly assigned to the experimental group/treatment. In case of experiments involving treatments such as nutrients manipulation/ drug administration, untreated and treated cells came from the very same culture and an homogeneous cell suspension was divided into many wells and either left untreated or subjected to the treatment.
Blinding	Investigators were blinded during data acquisition and analysis whenever possible (immunofluorescence and High Content Imaging). For WB experiments, blinding is not a standard in the field. However WB experiments were double checked by at least two researchers.

Reporting for specific materials, systems and methods

We require information from authors about some types of materials, experimental systems and methods used in many studies. Here, indicate whether each material, system or method listed is relevant to your study. If you are not sure if a list item applies to your research, read the appropriate section before selecting a response.

Materials & experimental systems

n/a	Involved in the study
☐	☒ Antibodies
☐	☒ Eukaryotic cell lines
☒	☐ Palaeontology and archaeology
☒	☐ Animals and other organisms
☒	☐ Clinical data
☒	☐ Dual use research of concern
☒	☐ Plants

Methods

n/a	Involved in the study
☒	☐ ChIP-seq
☒	☐ Flow cytometry
☒	☐ MRI-based neuroimaging

Antibodies

Antibodies used

The following antibodies were used in this study: phospho-p70 S6 kinase (Thr389) (1A5) (cat. # 9206 - 1:1,000), p70 S6 kinase (cat. # 9202 - 1:1,000), human TFEB (cat. # 4240 -1:1,000 WB/1:100 IF), TFEB-pS211 (E9S8N) (cat. # 37681 used at 1:1,000 WB/ 1:100 IF), Parkin (cat # 2132 1:500 WB/ 1:200IF); PINK1 (D8G3) (cat # 6946 1:1,000 WB), mTOR (7C10) (cat # 2983 1:200 IF), , GPNMB (E4D7P) XP (cat #38313 1:1000 WB)were from Cell Signaling Technology;
antibodies against GAPDH (6C5) (cat. no.sc-32233-1:15,000 WB), LAMP1 (H4A3) (cat #sc 200-11 1:400 IF), Galectin-3 (M3/38 sc-23938 1:800), 14-3-3 B-11 (sc-133232 1:1,000) were from Santa Cruz; Flag M2 (cat. #F1804 1:1,000 WB), ACTN2 EA-53 (cat. # A7811 dilution 1:1,000 IF) and actin AC-74 (# A2228 - 1:5,000 WB) were from Sigma Aldrich;
HA.11 epitope tag (cat. 901513 - 1:1,000) was from Biolegend; HA clone 3F10 (ref. 1186 7423001 1:800 FACS); Anti-HA High Affinity (ref.11867423001 1:500 IF) from Roche; Tomm20 clone 29 (cat # 612278 1:800 IF) and p62 Clone 3 (cat # 610832 1:800 WB) came from BD Biosciences. HRP-conjugated secondary antibodies for mouse (cat. # 401215 - dilution 1:5,000) and rabbit (cat. # 401315 - dilution 1: 5,000) were from Calbiochem; donkey anti-rabbit IgG (H + L) Alexa Fluor 488 (cat. #A-21206 - dilution 1:500), Alexa Fluor 568 (cat. # A-10042 - dilution 1:500), donkey anti-mouse IgG (H + L) Alexa Fluor 568 (cat. # A-10037 - 1:500), Alexa Fluor 647 (cat. # A-31571 - 1:500), Alexa Fluor 594 (cat. # A-21203 - 1:500), donkey goat anti-goat IgG (H + L) Alexa Fluor 647 (cat. # A-21447 - 1:500), donkey anti-rat IgG (H+L) Alexa Fluor 647 (cat- #A21247 1:500) were from Thermo Fisher Scientific.

Validation

The antibodies used in the study were bought from commercial vendors and were validated by the manufacturers and/or other studies. See individual antibody's web page (link shown below)on the manufacture's website for validation and relevant citations:
- mTOR: https://www.cellsignal.com/products/primary-antibodies/mtor- 7cl0-rabbit-mab/2983
- Phospho-p 70 S6 Kinase (Thr389): https://www.cellsignal.com/products/primary-antibodies/phospho-p70-s6-kinasethr389- la5-mouse-mab/9206
- p70 S6 Kinase: https://www.cellsignal.com/products/primary-antibodies/p70-s6-kinase-antibody/9202
- Human TFEB: https://www.cellsignal.com/products/primary-antibodies/tfeb-antibody/4240
-TFEB-pS211: https://www.cellsignal.com/products/primary-antibodies/phospho-tfeb-ser211-e9s8n-rabbit-mab/3 7681
-Parkin: https://www.cellsignal.com/products/primary-antibodies/parkin-anti body/2132
-PINK1: https://www.cellsignal.com/products/primary-antibodies/pinkl-d8g3-ra bbit-ma b/6946
- GAPDH (6C5): https://www.scbt.com/p/gapdh-antibody-6c5
- LAMP-1 (H4A3): https://www.scbt.com/p/lamp-1-antibody-h4a3
-Galectin3: https://www.scbt.com/it/p/galectin-3-antibody-m3-38
-TFE3: https://www.cellsignal.com/products/primary-antibodies/tfe3-antibody/14 779
-GFP: https://www.abcam.com/gfp-antibody-ab13970.html
-FLAG M2: https://www.sigmaaldrich.com/IT/it/product/sigma/f1804
- Actin: https://www.sigmaaldrich.com/cata1og/product/sigma/a2228
-HA.11 Epitope Tag: https://www.biolegend.com/en-us/products/anti-ha-11-epitope-tag-antibody-11071
-Anti-HA High Affinity: https://www.sigmaaldrich.com/IT/it/product/roche/roahaha
-Tomm20 :https://www.bdbiosciences.com/en-u s/products/reagents/microscopy-i maging-reagents/i mmunofluorescence-reagents/ purifled-mouse-anti-tom20.612278.

The antibodies used in this manuscript were chosen based on previous literature. Information about the validation of the primary antibodies used are accessible on the manufacturer's websites (reported above) along with references in which the antibodies were used/tested. The antibodies used are the most diffused and trusted in the field of the lysosomes, autophagy, and mTOR pathway analysis (Napolitano et al., Nature 2020. PMID: 32612235)

Eukaryotic cell lines

Policy information about cell lines and Sex and Gender in Research

Cell line source(s)

Hela and HK-2 cells were purchased from ATCC (Hela CCL/2; HK-2 CRL-2190).
FLCN-KO Hela and FLCN-KO HK-2 cell lines were generated in this study.

Human induced Pluripotent stem cell lines used in this study are:
hiPSCs, LUMC0020iCTRL-06 line (https://hpscreg.eu/cell-line/LUMCi028-A)
hiPSC (CBiPS1sv-4F-40, https://hpscreg.eu/cell-line/ESi007-A)

Authentication	KO cells were authenticated by WB or RT-PCR. Commercial cell lines were purchased recently from ATCC and validated by morphological analysis. hiPSCs were validated by PCR and by immunofluorescence analysis of stemness makers such as SSEA4 and TRA1-60.
Mycoplasma contamination	Cells were routinely tested and validated for the absence of mycoplasma.
Commonly misidentified lines (See ICLAC register)	No commonly misidentified lines were used.
